# Characterization of Phenolic Compounds in Wine Lees

**DOI:** 10.3390/antiox7040048

**Published:** 2018-03-25

**Authors:** Ye Zhijing, Amin Shavandi, Roland Harrison, Alaa El-Din A. Bekhit

**Affiliations:** 1 Department of Food Sciences, University of Otago, Dunedin 9054, New Zealand; Victor.Ye@lincolnuni.ac.nz; 2 Department of Wine, Food and Molecular Biosciences, Lincoln University, Lincoln 7647, Canterbury, New Zealand; Roland.Harrison@lincoln.ac.nz

**Keywords:** grapes, wine lees, antioxidant, total phenol, tannins, polymerization

## Abstract

The effect of vinification techniques on phenolic compounds and antioxidant activity of wine lees are poorly understood. The present study investigated the antioxidant activity of white and red wine lees generated at early fermentation and during aging. In this study, the total phenol content (TPC), total tannin content (TTC), mean degree of polymerization (mDP), and antioxidant activities of five white and eight red wine lees samples from different vinification backgrounds were determined. The results showed that vinification techniques had a significant (*p* < 0.05) impact on total phenol and tannin content of the samples. White wine lees had high mDP content compared with red ones. Catechin (50–62%) and epicatechin contents were the predominant terminal units of polymeric proanthocyanidin extracted from examined samples. Epigallocatechin was the predominant extension unit of white wine lees, whereas epicatechin was the predominant compound in red wine marc. The ORAC (oxygen radical absorbance capacity) assay was strongly correlated with the DPPH (α,α-diphenyl-β-picrylhydrazyl) assay, and the results showed the strong antioxidant activities associated with red wine lees (PN > 35 mg Trolox/g FDM) (PN: Pinot noir lees; FDM: Freeze-dried Material). This study indicates that tannin is one of the major phenolic compounds available in wine lees that can be useful in human and animal health applications.

## 1. Introduction

Approximately 75 million tonnes of grape were produced annually, of which 85% is used in the wine industry, and around 9 million tons of organic waste is generated [1]. These waste materials are high in organic substances which are recognized as environmental pollutants, since they have high chemical oxygen demand (COD) and biological oxygen demand (BOD) [2]. However, wine wastes (marcs, stalks, and dregs) of various grape varieties were reported to be high in phenolic content and their extracts exhibited strong biological activities [3,4]. Based on biological properties of phenolics and the increase of “sustainable” concerns, there is growing interest in the utilization of major wine wastes (vine stalk, grape skins, seeds, and pulps) including conversion of waste material into biofuels, nutrient supplements, food ingredients, and animal feed.

Wine lees consist of dead yeast, yeast residues or particles precipitated at the bottom of wine tanks or barrels [5]. Current management of this waste, such as discarding to a landfill or spreading in vineyards as compost, were reported to have a negative impact on soil [6]. Lees contribute about 14% of the total organic wastes produced in the wine [7], and a cost-effective use of this material should be of interest to the wine industry. However, only very few studies have used wine lees in useful applications, e.g., Hwang et al. [5] added wine lees to ice cream to improve the rheological and antioxidant properties of ice cream. In addition, wine lees is still poorly characterized and information on its potential biological activities are generally not available in the literature.

The present study examined the characteristics of lees from a variety of sources, including different grape varieties and winemaking techniques, in order to improve understanding of this material and devise useful strategies to add value to this waste stream. Lees samples were characterized by determining total phenolic content, tannin content, mean degree of polymerization (mDP), and antioxidant activity of their extracts.

## 2. Materials and Methods

### 2.1. Chemicals

Sodium carbonate, anhydrous di-sodium hydrogen orthophosphate, and ascorbic acid were from BDH (London, UK). Methylcellulose, gallic acid, and phloroglucinol were purchased from Sigma Chemical Co. (St. Louis, MO, USA). Trolox (6-hydroxy-2,5,7,8-tetramethylchroman-2-carboxylic acid), Folin–Ciocalteu reagent, α,α-diphenyl-β-picrylhydrazyl (DPPH), 2,2′-azobis(2-amidinopropane)dihydrochloride (AAPH), catechin, and epigallocatechin gallate were obtained from Sigma-Aldrich Chemical Co. (Steinheim, Germany). Sodium dihydrogen phosphate monohydrate was purchased from LabServ Biolab (Clayton, Australia). Ammonium sulfate was obtained from J.T. Baker (Philipsburg, PA, USA). Ethanol (100%) was obtained from Fisher Scientific (Loughborough, UK). Methanol (100%), acetic acid, and hydrochloride were obtained from Merck (Darmstadt, Germany). All reagents and chemicals used in this study were of analytical grade or higher. The water used was produced from E-pure Barnstead water system Model no. 02622. The water used was obtained from E-pure Barnstead water system Model no. 02622 (Thermo Scientific, Auckland, New Zealand).

### 2.2. Wine Lees and Sample Preparation

Wine lees used in this study were obtained from Lincoln University, (Canterbury, New Zealand) and commercial wineries during March–April 2009. A total of fourteen wine lees samples were obtained from small-scale winemaking exercises which utilized six white and seven red wine lees samples (sp. *Vitis vinifera*). The white varieties were Sauvignon Blanc (SB), Chardonnay (C), and Pinot Gris (PG), and the red variety was Pinot Noir (PN). Winemaking encompassed a number of common techniques, such as natural fermentation, commercial yeast inoculation, maceration on skins, pump over, and hand plunging (Table 1).

Lees samples were rotary evaporated at 40 °C for 20 min to remove ethanol, and the resultant material was frozen at −40 °C and then freeze-dried at a pressure of 0.5 mbar. Freeze-dried lees samples were ground with a mortar and pestle and kept in airtight containers at −20 °C until analysis.

Lees samples were extracted as described by Mercurio et al. [8]. Five mL of 50% (*v*/*v*) ethanol was added to 0.5 g of freeze-dried sample, and the sample was shaken using a thermostatic orbital shaker (Model OM 11, Ratek Instrument Ltd., Boronia, Australia) for 60 min. Samples were then centrifuged at 3000× *g* for 5 min using a Megafuge 1.0 with a Siehe rotor (Kendro Laboratory Products, Hamburg, Germany). Supernatants were decanted, subsampled and stored at −20 °C. Wine lees and sample preparation were done in triplicate.

### 2.3. Measurement of Total Phenolics Content

The total phenol content of wine lees extracts was determined as described by Makkar et al. [9]. A 0.1 mL aliquot of the wine lees extract was transferred to a labelled test tube followed by addition of 8.4 mL of deionized water and 0.5 mL of Folin–Ciocalteu reagent. After mixing, 1.0 mL of sodium carbonate solution (20% *w*/*v*) was added. The solution was mixed again and kept at room temperature for an hour. The absorbance of the mixture was read at 720 nm using a visible spectrophotometer (UNICAM Helios Alpha, Cambridge, England) against a water blank. This analysis was performed with triplicate measurements of three subsamples for each extract. The concentration of total phenolic was calculated from a standard curve constructed using gallic acid solutions following the method described above. The range of gallic acid standard concentration was between 0 and 1 g/L. The total phenolic content was expressed as mg gallic acid equivalents (GAE) per gram of dry lees (mg GAE/g FDM).

### 2.4. Measurement of Total Tannin Content

The total tannin content of extracts of freeze-dried wine lees was determined using the methylcellulose precipitable (MCP) tannin assay as described by Sarneckis et al. [10]. This is the Australian Wine Research Institute (AWRI) standard method for measuring the total tannin content of grape homogenates and red wines. The assay total volume was 1 mL for both treatment and control test. In treatment, 100 μL of extracts was added to a microcentrifuge tube followed by 300 μL of 0.04% methylcellulose (MC), 200 μL of saturated ammonium sulfate and 400 μL of distilled water (DI). The tubes were left at room temperature for 10 min, followed by centrifugation at 1430 g for 5 min using Biofuge 15 with #3743 Sepatech rotor (Heraeus, Hanau, Germany). The resultant supernatant was pipetted into a UV cuvette and the absorbance was read using a UV spectrophotometer at the absorbance of 280 nm. In the control test, there was no addition of MC, which was replaced by adding DI. Epicatechin was used for standard, and the range of epicatechin standard concentrations were 0, 25, 50, 75, and 100 μg/mL. For calculation of tannin content, the difference of the absorbance between control and treatment read at 280 nm was substituted in the linear equation generated from the epicatechin standard curve. The results were corrected for any dilutions by using the appropriate dilution factor. The tannin content was then reported on epicatechin equivalent basis (mg/L epicatechin eq).

### 2.5. Phloroglucinol Analysis

The mean degree of polymerization (mDP) of wine lees extracts was determined as described by Kennedy and Jones (2001) [11]. The method is based on the acid-catalyzed cleavage of proanthocyanidin in the presence of excess phloroglucinol followed by chromatographic analysis of the products. One gram of wine lees extract was transferred to a pre-weighed round bottom flask (RBF) and 3 mL of deionized water was added. The volume was reduced to 0.2–0.3 g by rotary evaporation at 40 °C to remove ethanol, followed by addition of 3 mL of deionized water. A Sep-Pak cartridge (C18#WAT051910, Global Science) was pre-activated with 5 mL methanol (HPLC grade) followed by 7.5 mL ethyl acetate and 7.5 mL deionized water, and finally dried with oxygen-free nitrogen gas with a flow rate of 1 L/min for an hour. Extracts were then passed through the activated Sep-Pak column using a syringe. The dried Sep-Pak column was flushed with 5 mL ethyl acetate, which was eluted to waste. Five mL of HPLC grade methanol was used to elute proanthocyanidins from the Sep-Pak cartridge into a clean pre-weighed RBF. The methanol extract was reduced to less than one gram by rotary evaporation at 40 °C and made up to a total weight of one gram with methanol. The extract was then transferred to a 2 mL microcentrifuge tube, and stored at −20 °C for acid-catalyzed cleavage. The extracts from each subsample were analyzed in duplicate.

For acid catalysis, a 0.50 mL aliquot of blank reagent (0.2 M HCl in methanol containing 20 g/L ascorbic acid) or phloroglucinol solution (0.2 M HCl in methanol containing 100 g/L phloroglucinol and 20 g/L ascorbic acid) was added to 0.50 mL labelled control and phloroglucinol test tubes, respectively. All the samples and controls were incubated at 50 °C for 20 min, and the reaction was stopped by adding 5 mL of 40 mM aqueous sodium acetate solution prepared fresh [11].

**Figure 1:**
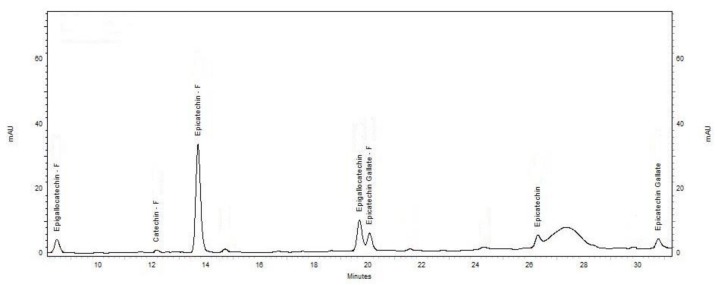
The chromatograms of HPLC profile.

An Agilent HPLC 1100 series was used to analyze phloroglucinol adducts according to the method described by Kennedy and Jones (2001) [11]. The column was a Phenomenex Luna C18 (5 µm, 250 × 4.60 mm), protected by a C18 column guard. The column temperature was 25 °C and the running time for each injection was 42 min. Two mobile phases (A and B, containing 2% *v*/*v* aqueous acetic acid and 2% *v*/*v* acetic acid in methanol, respectively) were used and the eluting peaks were monitored at 280 nm. The flow rate was set at 0.8 mL/min, with the mobile phase set initially as 95% mobile phase A/5% mobile phase B for 5 min, followed by 90% mobile phase A/10% mobile phase B for 25 min, then 60% mobile phase A/40% mobile phase B for 30 s, and finally, 100% mobile phase B for 6 min, before equilibrating the column to the initial running conditions. To construct the standard curve, solutions A (1000 ppm catechin solution) and B (1000 ppm epicatechin solution) were made and used at concentrations of 0 to 100 ppm. The standard samples were processed as described above. To estimate the proanthocyanidin adducts, the response factors stated by [11] were used. The molar sum of all flavan-3-of monomer and phloroglucinol adducts was divided by the molar sum of all flavan-3-ol monomers to calculate mDP. Figure 1 shows an example of the HPLC chromatogram obtained in this study.

### 2.6. Antioxidant Activities

Antioxidant activity of wine lees extracts was determined using α,α-diphenyl-β-picrylhydrazyl radical (DPPH•) and oxygen radical antioxidant capacity (ORAC) methods.

The ability of lees extracts to scavenge the DPPH• was evaluated using methods previously described by Sánchez-Moreno et al. [12], with modification. Each lees extract was serially diluted using 50% ethanol solution to produce concentration replacing 50, 25, 12.5, 6.25, 3.13, and 1.56% of the original extracts. A 75 μL aliquot of each diluted sample was added to 2925 μL of 0.025 g/L DPPH• solution (prepared in 100% HPLC Grade methanol) in a 3 mL visible cuvette, and mixed by gentle inversion. DPPH• solutions were prepared daily and kept on ice and in a light-tight container all the time. The absorbance at 515 nm (A_515_) was measured at 0, 5, 10, 20, 30, and 60 min of reaction using a spectrophotometer against a water blank. The cuvettes were covered with parafilm and kept in a light-tight container between absorbance measurements.

The concentration of remaining DPPH• in the reaction medium at each reaction time was calculated from a calibration curve of DPPH• of absorbance at 515 nm (A515) against concentration. The equation below was applied to calculate the percentage of remaining DPPH• at the reaction time of 10 min. The percentage of remaining DPPH• was then plotted against weight (mg) of extract.

% DPPH•R = [(DPPH•)T/(DPPH•)T = 0] × 100

where (DPPH•)T is the DPPH• concentration at a plateau; (DPPH•)T = 0 is the DPPH• concentration at time zero.

Results were also presented in EC_50_ (Effective concentration, the amount of sample required to decrease the initial concentration of DPPH• by 50%). EC_50_ of different sample extracts were calculated from the regression equation.

The ORAC assay is a widely used method to measure the antioxidant activity in medical and life sciences fields. It uses 2,2′-azobis(2-amidinopropane)dihydrochloride (AAPH) as a peroxyl radical generator [13], and reports the antioxidant activity of a compound relative to a standard antioxidant (Trolox). The antioxidant capacity of lees extracts was determined using ORAC assay according to Hwang et al. [5], with modifications. All determinations were performed on three subsamples and the measurements were in triplicate.

FLUOstar Omega multifunctional microplate reader (BMG LABTECH, Orthenberg, Germany) was used to measure the fluorescein intensity. The instrument was set up to read fluorescence intensity mode. The excitation, emission, and cut off wavelength were 485 nm, 538 nm, and 530 nm, respectively, and the gain was adjusted to 85%. The measurement was taken over 60 min at 1 min intervals.

Diluted antioxidant standard and samples (25 μL) were added to the 96-well microplate, followed by the addition of 150 μL of 10 nM fresh fluorescein working solution to each well. The microplate was then covered by parafilm and incubated at 37 °C for 30 min. After incubation, 25 μL of the AAPH (65 mg/mL) solution was automatically injected into each well by the plate reader pump followed by 5 s shaking.

The area under the curve (AUC) method was used for quantification of ORAC (Huang et al., 2002). AUC of blank and antioxidant was calculated from the equation below:

$$\mathrm{AUC}=1+\overset{60}{\underset{i=1}{\sum }}\frac{\mathrm{RFUt}=i}{\mathrm{RFU}0}$$

where RFU0 is the relative fluorescence value of time point zero; RFUt is relative fluorescence value of time points. The net AUC can be calculated by subtracting AUC (blank) from AUC (antioxidant). Based on the standard concentration between 0 and 0.05 mg/mL Trolox, the antioxidant activity was calculated and expressed in equivalent Trolox concentration (TE).

### 2.7. Statistical Analysis

Analysis of variance (ANOVA) was carried out to investigate the effects of samples (dependent variable) on the measured parameters. Significant difference at *p*-value < 0.05 was tested by application of Tukey’s multiple comparisons test. Error bars in graphs indicate standard deviation (SD) of the mean. Pearson’s correlation coefficients among the measured parameters were also calculated. The analysis was Genstat^®^ Software (Version 9.0, VSNI, Hemel Hempstead, UK) was used for statistical analysis.

## 3. Results and Discussion

### 3.1. Total Phenol Content (TPC) and Total Tannin Content (TTC)

**Figure 2:**
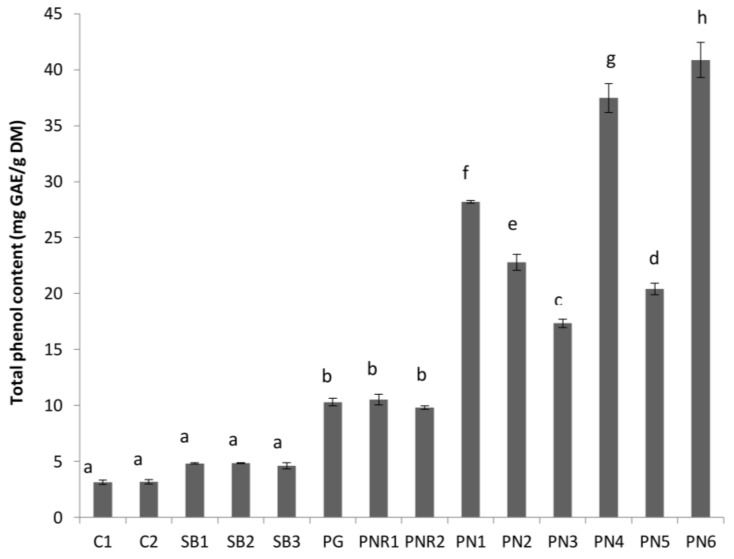
Total phenol content in mg gallic acid equivalents (GAE) per gram of dry lees (mg GAE/g FDM) of different wine lees extracts investigated in the present study. Treatments do not share the same letter (a–h) are significantly different (*p* < 0.05). Error bars are the standard deviation of replicate analysis.

The average TPC of the wine lees extracted with 50% ethanol is shown in Figure 2. The TPC of Pinot Noir (PN) lees (ranged between 17.3 ± 0.4 and 40.9 ± 1.6 mg/g FDM) were significantly higher than PN rosé (9.8 ± 0.2 and 10.5 ± 0.5 mg/g FDM) and white wine lees (ranged between 3.1 ± 0.2 and 10.3 ± 0.4 mg/g FDM). This variation in TPC can be attributed to differences in vinification and the processing steps during the winemaking process. The contact time between wine and grape solids (seeds and skin), the major source of phenolics in wine and lees, follows the order red wine > rosé > white wine, and can greatly affect the mass transfer of phenolics from grape solids to wine, and consequently, to lees. This vinification and the processing steps also affect TPC within the same wine type. For example, the TPC of Pinot Gris (PG) lees was significantly greater (*p* < 0.05) than lees from other white varieties (C1, C2, SB1, SB2, and SB3). This may be explained by the processing of PG wine where a maceration step is used during processing (Table 1). In addition, PG grapes may also contain more anthocyanins and other phenolic compounds in their skins, as they are more coloured compared to C and SB varieties, and therefore, may contain more. A maceration step with skins during the winemaking of PG will increase the mass transfer of the phenolics from the skin to the wine and wine lees.

The TPC of lees from rosé wine (PNR1 and PNR2) was not affected by pre-fermentation maceration period (1 week and 48 h for PNR1 and PNR2, respectively). This is consistent with the results of Sacchi et al. [3], which demonstrated that cold maceration alone has no effect on the phenolic content of wine. The TPC of PG was not different (*p* > 0.05) from both PNR1 and PNR2.

The TPC of red wine lees varied widely (P < 0.05) depending on the processing conditions and geographical location of the samples. PN lees from Central Otago with no cap management (PN6) had the highest TPC (41.0 ± 1.6 mg/g FDM), which was more than twice the level found in Waipara Pinot Noir macerated for 1-week pre-fermentation (PN3, 17.3 ± 0.4 mg/g FDM). A “cap” is formed when crushed grapes are pushed to the surface of must by yeast-generated carbon dioxide during fermentation. “No cap management” means a cap was allowed to form, and the cap formation was not disturbed, which would allow trapping the heat generated during the fermentation process and an increase in the ferment temperature would result. The rise of fermentation temperature (above 15 °C) is advantageous to the growth of *Saccharomyces cerevisiae* [14], and can speed the winemaking process. Our results are in agreement with the findings of Sacchi et al. [3] for red wine, where a higher fermentation temperature led to a higher TPC in PN wine.

The effects of maceration on PN is observed in red wine lees samples in which PN3 with 1-week pre-fermentation maceration had a lower TPC than PN1 (no maceration). Pinelo et al. [15] reported that maceration alone has a negative effect on TPC in PN wines. However, the presence of ≥ 50 mg/L of sulfur dioxide increases the TPC in wine. This may explain the observed lower TPC in sample PN3.

The technique used for increasing the release of phenolics in wine may have an impact on the TPC in lees. For example, Central Otago PN lees from pumped over wine (PN2) had higher TPC (*p* < 0.05) compared to hand plunged (PN5) technique. This observation is consistent with Sacchi et al. (2005) that varying TPC was obtained in the wine that is treated with “pumped over” compared with “hand plunged” techniques. Moreover, the addition of oak wood chips (PN4) may lead to a high TPC in wine lees. Therefore, the TPC of wine and any by-products (such as lees in the present case) will vary depending on the vinification techniques used, and the presence of certain chemicals, such as sulfur dioxide [3].

Based on the results of this study and previous literature [16,17], the TPC in wine wastes is affected by grape varieties, growing conditions (location of the vineyard), and the vinification techniques used.

### 3.2. Total Tannin Content (TTC)

**Figure 3:**
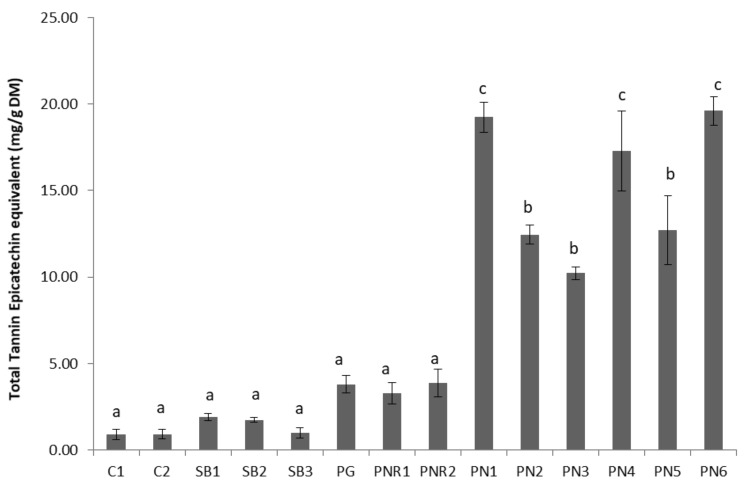
Total tannin content (epicatechin equivalent/g FDM) of different wine lees extracts. Treatments which do not share a letter (a–c) are significantly different (*p* < 0.05). Error bars are the standard deviation of replicate analysis.

Total tannin content (TTC) of the wine lees samples is shown in Figure 3. The results for TTC are expressed as mg epicatechin equivalent per g of freeze-dried material. The pattern of total tannins content was similar to that found in TPC. Based on the TTC content, the lees samples can be divided into three groups; low (white wine lees), medium (PG and PNRs lees), and high (PN red wine lees). There were no significant differences between TTC of white wine lees and PNRs lees (*p* > 0.05). However, more tannins were obviously present in red lees compared with white and rosé wines lees (Figure 2). The similarity of TTC of both PNR1 and PNR2 may suggest that incubation time has no significant effect on lees TTC. Within PNs, there were no significant differences between PN1, PN4, and PN6, and the TTC of PN2, PN3, and PN5 were very similar (Figure 3). The TTC of PN1 was significantly greater than PN3 (*p* < 0.05) maybe because the tannin extractability in PN3 was limited by applying 1 week pre-fermentation maceration with an insufficient amount of SO_2_. TTC of PN6 was significantly greater than PN2 and PN5 (*p* < 0.05). This may indicate that rise of temperature during fermentation allowed the extraction of more tannins than pump over (PN2) and hand plunged (PN5) techniques. The high TTC of PN4 might be due to the contribution of oak chips added in maceration. According to Gao et al. [18], the extraction of tannin from the grape is limited by its solubility, however, this limitation can be overcome by increasing alcohol content, use of sulfur dioxide, and increasing the fermentation temperature and skin contact time [3]. Therefore, PN1 might be fermented with a higher temperature, which dramatically increased the extraction of tannins from crushed grapes. Gallic acid is the fundamental structural unit of hydrolysable tannin which is found in considerable amounts in oak chips or barrels [16]. Therefore, the lees from the red wine either without application of extraction methods or with oak chips involved in maceration is a good source of tannin.

### 3.3. Polymeric Tannin Profile in Wine Lees

**Figure 4:**
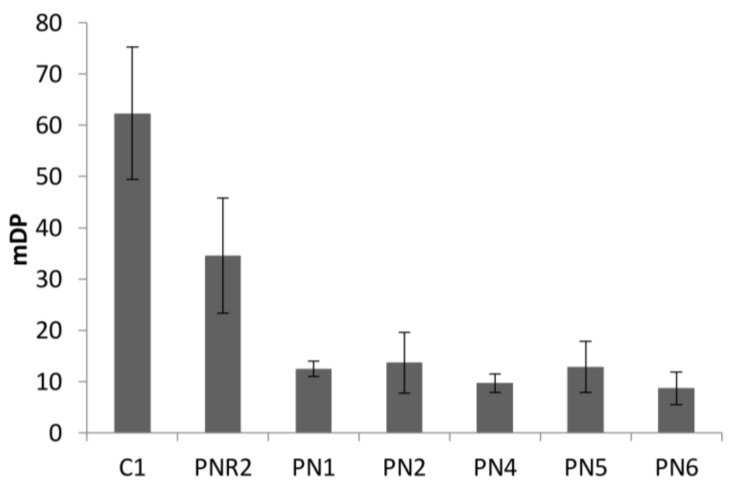
Mean degree of polymerization of eight wine lees extracts.

The mean degree of polymerization (mDP) of proanthocyanidins extracted from seven wine lees sample was determined by phloroglucinol analysis. The mDP values of red wine lees were low, ranging from 8.7 ± 3.2 to 12.7 ± 5.0 (Figure 4). By contrast, the C1 lees sample possessed the much higher mDP of 62.3 ± 12.9, which might be attributed to the source of proanthocyanidin, oxidation, and anthocyanin in red wine. In white winemaking, grape skin is the only source of proanthocyanidin, and grape skin and seeds are not contact with must or wine. By contrast, both skin and seeds proanthocyanidin are extracted to red wine as well as anthocyanin. Cosme et al. [19] reported that the mDP value of proanthocyanidins in the skin (3.8 to 81.0) was higher than in seeds (2.8 to 12.8). During the wine aging, oxidation generated polymeric proanthocyanidin in wine. However, the presence of anthocyanin in red wine limited the size of the polymer, due to the anthocyanin–tannin interaction [20]. PNR2 lees which had an mDP of 34.6 ± 11.2, and PN lees had the lowest mDP value range from 8.7 to 12.5. This is consistent with the levels reported for white, rosé, and red wines [21]. The differences of mDP values between lees may be caused by polymerization of flavan-3-ol monomers during the aging process of winemaking.

The monomer composition (%) of terminal and extension units in the breakdown of polymeric proanthocyanidin were calculated (Table 2). The results showed that catechin (50–62%) was the predominant terminal unit of proanthocyanidin extracted from both white and red wine lees. Epicatechin was also a major terminal lees. By contrast, epigallocatechin was the predominant extension unit of white wine lees and epicatechin was predominant in red wine lees.

Bordiga et al. [22] and Lee [23] reported structural characteristics of polymeric skin and seed proanthocyanidins of two red wines and four white wines. Epigallocatechin was not present in the terminal unit in the extracts of skin, seed, marc, and lees proanthocyanidins generated from both white and red winemaking. The structural characteristic of the terminal units of polymeric proanthocyanidin of lees in the present study was in agreement with those found in skin [22], but the extension units were different in that epicatechin was the predominant extension units of white wine lees and epigallocatechin was predominant in red lees. The structural characteristic of polymeric seed proanthocyanidin is different from skins and lees, where catechin, epicatechin, epicatechin gallate had similar contents of the terminal units in the extract from both red and white wine by-products; and epicatechin was the predominant extension unit in seed [22]. Similar results in wine were also reported by Lee et al. [23].

### 3.4. Antioxidant Activity

The antioxidant activities of the wine lees extracts were evaluated through their capacity to scavenge DPPH and AAPH radicals (Table 3). EC_50_ is a measurement of capacity to scavenge DPPH radicals which are expressed as the amount of dry lees required to scavenge 1 g DPPH radicals. Extracts of red wine lees exhibited the highest activity (EC_50_ ranged from 12.6 to 17.7 g FDM/g DPPH), followed by PNRs, and PG (EC_50_ ranged from 33.6 to 35.9 g FDM/g DPPH), and white wine lees extracts exhibited the least activity (EC_50_ ranged from 40.6 to 78.2 g FDM/g DPPH). The DPPH• data are also presented as a percentage of DPPH remaining over 60 min of reaction to demonstrate the efficacy of scavenging the radical (demonstrated by the rate of scavenging rather than EC_50_, which shows the capacity of scavenging). About 15 mg of Chardonnay lees was required to reduce DPPH radicals in the assay by 50% within ten minutes. The same level of inhibition was achieved after 20 min with 7.50 mg of lees. Only 7.50 mg of SB samples, 3.75 mg lees of PG and PNRs, were required to achieve 50% reduction within ten minutes. PN wine lees showed a much stronger free radical scavenging activity, with only 0.938 mg of samples required to achieve 50% reduction. While the scavenging of DPPH radicals was concentration-dependent, PN lees showed a different behaviour where the percentage of DPPH remaining at 7.50 mg of lees was higher than the ones with 1.875 and 3.750 mg of lees. The higher amount of lees addition leads to an early termination of the reaction. Villaño et al. [24] reported the same observation and pointed out that this change in the reaction of polyphenolic compounds towards DPPH free radical is due to phenols being under pseudo-first-order conditions. It is therefore recommended that DPPH scavenging assay be monitored over the reaction period and to avoid measurements after a fixed incubation time, as the latter case can generate erroneous results.

While sample PN4 had lower TPC and TTC than PN6, a similar level of antioxidant activity was found in these two samples. As mentioned earlier, maceration with oak chips for samples PN4 can lead to higher gallic acid content which can change the composition of the phenolics in the extracts without a significant increase in the TPC. Larrauri et al. [25] and Villaño et al. [24] reported that gallic acid has a high antioxidant activity (EC_50_ = 0.026 g/g DPPH) compared with many other phenolic compounds. Therefore, lees macerated with oak chips will have higher gallic acid content and have a better antioxidant activity similar to wine aging in oak barrel or macerated with oak chips.

**Figure 5:**
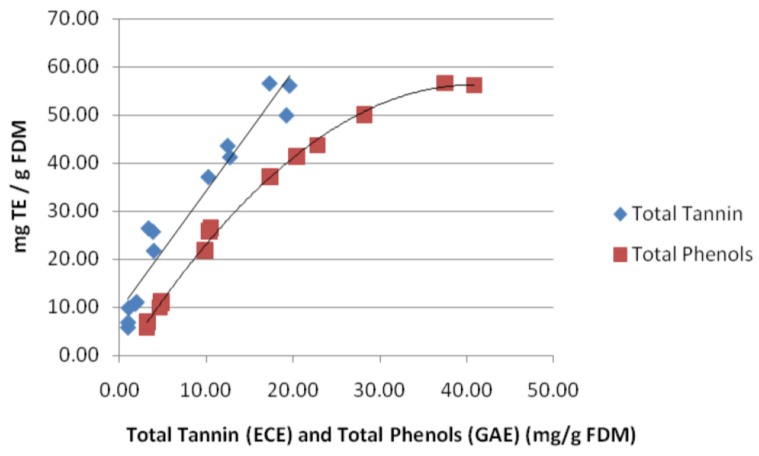
The relationship between the oxygen radical antioxidant capacity of total tannin content (TTC) and total phenol content (TPC) of wine lees.

The results of ORAC assay was expressed as the capacity of equivalent Trolox (mg TE/g FDM) to scavenge AAPH radicals. The results are in agreement with the results of DPPH radicals scavenging activity, where PN1, PN4, and PN6 showed the highest antioxidant activity. Figure 5 shows the relationship between free radical scavenging capacity and TTC and TPC of wine wastes. Results are easily divided into three groups, low (white wine lees, approximately 10 mg Trolox/g FDM), intermediate (PG and PNRs, 20 < TE < 30 mg Trolox/g FDM), and high (PNs, >35 mg Trolox/g FDM). The ORAC assay was strongly correlated to TPC and TTC, indicating that most of the antioxidant activities observed were related to TPC and TTC of lees. Moreover, a good linear relationship was found between antioxidant activities and TTC in which TTC is proportional to antioxidant activity. A different relationship of TPC and antioxidant activity was obtained that showed that TPC was proportional to antioxidant activity when TPC was less than 30 mg GAE/g FDM, and the increase of antioxidant activity was reduced when TPC was greater than 30 mg GAE/g FDM. These results may indicate that tannin is one of the major phenolic compounds that contribute free radical scavenging abilities. The strong correlations were also found between DPPH and TPC and TTC (Table 4). The antioxidant activity (EC_50_) of lees extracts (PN) in current study ranged from 4361.1 ± 20.3 to 6225.1 ± 96.2 mg extract/g DPPH. In comparison with the finding in PN and Pinot Meunier (PM) wine wastes reported by Cheng (2011), the antioxidant activity (EC_50_) of PN lees extracts are similar or higher than skin (EC_50_ ranged from 616.4 to 9606), and lower than pomace and seed (EC_50_ ranged from 616.4 to 9606, 190.9 to 1753.1, and 153.3 to 225.9 mg extract/g DPPH, respectively).

## 4. Conclusions

Results from the present study show that PN wine lees contained high phenolic compounds and antioxidant activities, and therefore, it is a useful source of antioxidants. The vinification techniques can affect the TPC and TTC, and consequently, their biological activities. In addition, the results may indicate that tannin is one of the major phenolic compounds that contribute free radical scavenging abilities.

The mDP values of white wine lees were higher than red ones, and this might be attributed to the source of proanthocyanidin, oxidation, and anthocyanin in red wine. Catechin was one of the predominant terminal units of polymeric proanthocyanidin found in both white and red wine lees. Epicatechin was also a major terminal unit of polymers in lees. Epigallocatechin and epicatechin were the predominant extension units of white and red wine lees, respectively. The above results indicate that red wine lees is a very rich in phenolics with high antioxidant activities, and can potentially be an easier material to extract phenolics from, unlike grape seed and skin, which require several drying and size reduction processes before extraction. Lees has a great potential to be used as a source of bioactive compounds.

## Figures and Tables

**Table 1:** Description of lees samples.

Sample Code	Sample Description
C1	Lincoln University Chardonnay 1
C2	Lincoln University Chardonnay 2
SB1	Lincoln University Sauvignon Blanc, natural yeast ferment 1
SB2	Lincoln University Sauvignon Blanc, natural yeast ferment 2
SB3	Lincoln University Sauvignon Blanc, inoculated with commercial yeast
PG	Lincoln University Pinot Gris HB clone, macerated on skins
PNR1	Lincoln University Pinot Noir Rosé, 1-week pre-ferment maceration
PNR2	Lincoln University Pinot Noir Rosé, 48 h pre-ferment maceration
PN1	Waipara Pinot Noir, no pre-ferment maceration
PN2	Central Otago Pinot Noir, pumped over
PN3	Waipara Pinot Noir, 1-week pre-ferment maceration
PN4	Waipara Pinot Noir, 5 days pre-fermentation maceration with oak chips
PN5	Central Otago Pinot Noir, hand plunged
PN6	Central Otago Pinot Noir, No cap management

**Table 2:** Percentage of monomers in the breakdown of the phenolic in polymer fractions.

Sample	Terminal Units (No PG) (%)				Extension Units (PG) (%)			
	C	EC	ECG	EGC	C	EC	ECG	EGC
White wine lees								
C1	50.7	38.0	11.3	0.0	0.0	15.8	0.0	84.2
PG	52.9	37.1	8.1	0.0	0.9	51.3	2.1	45.6
Rosé wine lees								
PNR2	56.8	40.6	8.6	0.0	1.1	59.2	2.8	36.9
Red wine lees								
PN1	59.8	35.9	4.4	0.0	1.5	74.8	1.8	22.0
PN2	55.1	36.2	8.7	0.0	1.4	63.4	4.6	30.7
PN4	55.9	36.4	7.1	0.0	1.2	63.2	2.8	32.8
PN5	61.6	48.4	10.5	0.0	1.3	63.0	3.4	32.4
PN6	58.1	41.8	8.7	0.0	1.2	61.8	3.0	34.0

C: catechin; EC: epicatechin; ECG: epicatechin gallate; EGC: epigallocatechin; PG: phloroglucinol adduct.

**Table 3:** Summary of antioxidant activity of wine lees extracts.

Sample	EC_50_ (mg Lees Extracts/g DPPH)	ORAC (mg Trolox/g FD Material)
C1	12,166.3 ± 728.9	5.8 ± 0.10
C2	12,608.3 ± 870.7	6.9 ± 0.74
SB1	13,780.7 ± 895.4	11.1 ± 0.41
SB2	15,030.0 ± 847.6	10.8 ± 0.17
SB3	7803.3 ± 237.5	9.9 ± 0.39
PG	6724.1 ± 80.8	25.8 ± 1.70
PNR1	6548.6 ± 1180.7	26.5 ± 3.65
PNR2	6996.9 ± 323.4	21.8 ± 4.10
PN1	5090.4 ± 56.6	50.0 ± 3.12
PN2	5490.4 ± 279.0	43.7 ± 1.53
PN3	7150.8 ± 230.5	37.2 ± 0.19
PN4	5009.6 ± 48.5	56.6 ± 2.77
PN5	6262.0 ± 177.9	41.3 ± 0.81
PN6	5332.8 ± 20.2	56.2 ± 3.39
Trolox	104.5 ± 6.4	-
Gallic acid	58.2 ± 3.2	-

**Table 4:** Pearson’s coefficient correlations between TPC, TTC, mDP, and antioxidant activity (DPPH and ORAC) of extracts obtained from different wine lees using 50% extraction solvent of ethanol. The values in brackets are the significance of the correlations.

Correlation	TPC	TTC	DPPH	ORAC
TTC	0.955			
	(0.000)			
DPPH	−0.808	−0.819		
	(0.000)	(0.000)		
ORAC	0.959	0.954	−0.894	
	(0.000)	(0.000)	(0.000)	
mDP	−0.404	−0.464	0.354	−0.371
	(0.050)	(0.022)	(0.089)	(0.075)
